# Development and internal validation of a nomogram for predicting adverse pregnancy outcomes in women with early-onset preeclampsia

**DOI:** 10.3389/fmed.2026.1828294

**Published:** 2026-06-17

**Authors:** Haiqiong Ye, Qinlin Zheng

**Affiliations:** Department of Obstetrics, The Affiliated Hospital, Southwest Medical University, Luzhou, China

**Keywords:** adverse pregnancy outcomes, early-onset preeclampsia, nomogram, prediction model, risk factors

## Abstract

**Background:**

Early-onset preeclampsia (EOPE) is a severe obstetric complication associated with a high risk of adverse maternal and fetal outcomes. Early identification of patients at high risk of adverse pregnancy outcomes may facilitate timely clinical intervention and improve pregnancy management. This study aimed to develop and internally validate a clinical prediction model for adverse pregnancy outcomes in women with EOPE.

**Methods:**

This retrospective observational study included 425 women diagnosed with EOPE who were admitted to a tertiary teaching hospital in Southwest China between January 2018 and December 2025. The composite adverse pregnancy outcome was defined as at least one predefined maternal or fetal/neonatal adverse event occurring after admission, excluding baseline pregnancy-related complications used as candidate predictors. Patients were randomly divided into a training cohort (*n* = 298) and a validation cohort (*n* = 127). Candidate predictors were restricted to variables measured or diagnosed at admission or within the first 24 h after admission, before the occurrence of the composite adverse pregnancy outcome. Candidate predictors were screened using least absolute shrinkage and selection operator (LASSO) regression and further evaluated using multivariable logistic regression. Independent predictors were used to construct a nomogram-based prediction model. Model performance was evaluated in terms of discrimination, calibration, and clinical utility using receiver operating characteristic (ROC) curves, calibration curves, and decision curve analysis (DCA), with internal validation performed in the split-sample validation cohort.

**Results:**

Seven predictors were independently associated with the composite adverse pregnancy outcome and were incorporated into the nomogram: gestational age at admission, systolic blood pressure, urine protein score, platelet count, aspartate aminotransferase (AST) level, fetal growth restriction, and hemolysis, elevated liver enzymes, and low platelet syndrome (HELLP) syndrome. In clinically interpretable units, lower gestational age at admission, each 10-mmHg increase in systolic blood pressure, each 1-grade increase in urine protein score, each 50 × 10^9^/L decrease in platelet count, each 10-U/L increase in AST, fetal growth restriction, and HELLP syndrome were associated with increased risk. The model showed good discrimination, with an area under the curve (AUC) of 0.859 (95% CI: 0.754–0.897) in the split-sample validation cohort. Calibration curves showed acceptable agreement between predicted and observed risks, and decision curve analysis suggested potential clinical net benefit across clinically relevant threshold probabilities.

**Conclusion:**

We developed and internally validated a nomogram-based prediction model for adverse pregnancy outcomes in women already diagnosed with EOPE. The model is intended for admission-based risk stratification after EOPE diagnosis, rather than for early prenatal prediction, population screening, or prediction of the initial occurrence of EOPE. The model demonstrated good predictive performance in this single-center cohort and may support clinical risk stratification after external validation. The proposed risk categories are exploratory and should not be used as definitive clinical decision cutoffs without external validation. Independent multicenter validation is required before clinical implementation.

## Introduction

Preeclampsia is a major hypertensive disorder of pregnancy and remains a leading cause of maternal and perinatal morbidity and mortality worldwide (1). It is estimated to affect approximately 2–8% of pregnancies globally and contributes substantially to adverse maternal and fetal outcomes, particularly in low- and middle-income countries (2). Among its subtypes, early-onset preeclampsia (EOPE), defined as preeclampsia occurring before 34 weeks of gestation, is considered a more severe clinical phenotype and is associated with higher risks of maternal complications, fetal growth restriction, preterm delivery, and perinatal death (3). The pathophysiology of EOPE is complex and involves abnormal placentation, endothelial dysfunction, systemic inflammation, and multiorgan involvement (4). Because EOPE often progresses rapidly and can lead to severe maternal and fetal complications, early identification of patients at high risk of adverse pregnancy outcomes is essential for optimizing clinical management and improving pregnancy prognosis (5). More broadly, early identification of women at increased risk of preeclampsia before or during early pregnancy is clinically important because it may allow timely preventive strategies, including low-dose aspirin prophylaxis in high-risk pregnancies. Evidence from randomized trials and meta-analyses suggests that low-dose aspirin can reduce the risk of preeclampsia when used appropriately in women at increased risk (6). Once EOPE has developed, risk stratification remains essential for identifying patients at high risk of subsequent adverse maternal and fetal/neonatal outcomes and for guiding the intensity of surveillance, referral, and individualized obstetric management.

In recent years, considerable efforts have been made to identify clinical and biochemical predictors associated with adverse pregnancy outcomes in women with preeclampsia. Previous studies have reported that several maternal characteristics and laboratory indicators, such as gestational age at diagnosis, blood pressure levels, platelet count, liver enzyme levels, and fetal growth restriction, are associated with disease severity and pregnancy prognosis (7–9). However, most existing studies have focused on individual risk factors rather than integrating multiple predictors into a clinically applicable prediction model. Moreover, many previously reported models lack sufficient validation or are difficult to apply in routine clinical practice due to the use of complex biomarkers or advanced imaging techniques (10, 11). Therefore, there remains a need for practical and reliable predictive tools based on routinely available clinical variables to assist clinicians in risk stratification and decision-making for patients with EOPE.

Clinical prediction models and nomograms have been increasingly used in medical research to provide individualized risk estimation and facilitate clinical decision-making (12). By integrating multiple predictors into a quantitative risk assessment tool, such models can improve the accuracy of prognostic evaluation and help guide personalized clinical management. Nevertheless, prediction models specifically designed for assessing adverse pregnancy outcomes in patients with EOPE remain limited, particularly those developed using routinely available clinical data and validated for predictive performance (13).

Therefore, the present study aimed to develop and internally validate a clinically applicable prediction model for adverse pregnancy outcomes in women already diagnosed with EOPE at admission. The model was designed for admission-based risk stratification after EOPE diagnosis, rather than for early prenatal prediction, population screening, or prediction of the initial occurrence of EOPE. Using routinely collected clinical and laboratory variables, we applied LASSO regression for feature selection and constructed a multivariable logistic regression model to establish a nomogram for individualized risk prediction. The predictive performance of the model was evaluated in terms of discrimination, calibration, and clinical utility.

## Methods

### Study design

This study was designed as a retrospective observational study aimed at developing and internally validating an admission-based clinical prediction model for adverse pregnancy outcomes among patients already diagnosed with early-onset preeclampsia (EOPE). The model was not intended for early prenatal prediction, population screening, or prediction of the initial occurrence of EOPE. The study followed the principles recommended by the Transparent Reporting of a Multivariable Prediction Model for Individual Prognosis or Diagnosis (TRIPOD) statement to ensure methodological transparency and reproducibility. The overall analytical framework included variable selection using least absolute shrinkage and selection operator (LASSO) regression, multivariable logistic regression modeling, construction of a nomogram, and evaluation of model performance through discrimination, calibration, and clinical utility analyses.

### Setting

This study was conducted at a tertiary teaching hospital in Southwest China that provides comprehensive obstetric care and serves as a regional referral center for high-risk pregnancies. Clinical data were extracted from the hospital’s electronic medical record system. The study period extended from January 2018 to December 2025, during which consecutive patients diagnosed with early-onset preeclampsia were identified and screened for eligibility.

### Participants

Eligible participants were pregnant women diagnosed with early-onset preeclampsia who were admitted to the hospital during the study period. Early-onset preeclampsia was defined according to established clinical guidelines as preeclampsia occurring before 34 weeks of gestation, characterized by new-onset hypertension after 20 weeks of gestation accompanied by proteinuria or other systemic manifestations. Patients were included if they had documented pregnancy outcomes and available data for all candidate predictors required for model development. Patients were excluded if they had multifetal pregnancies, preexisting severe systemic diseases that could substantially affect pregnancy outcomes, including documented autoimmune diseases requiring long-term systemic treatment before or during early pregnancy, incomplete medical records, or missing key predictor or outcome data. After applying these criteria, the final analytic dataset included 425 eligible patients. We further assessed the completeness of all candidate predictors and the composite adverse pregnancy outcome in the final analytic dataset; no missing values were observed for any included variable. The dataset was randomly divided into a training cohort (*n* = 298) for model development and a validation cohort (*n* = 127) for internal validation using a ratio of approximately 7:3.

### Variables

The primary outcome of interest was a composite adverse pregnancy outcome, defined as the occurrence of at least one predefined maternal or fetal/neonatal adverse event after admission and during hospitalization, delivery, or the early neonatal period, as documented in the medical records. The selection and definition of outcome components were based on established obstetric and neonatal criteria and were informed by authoritative guidance on hypertensive disorders of pregnancy and neonatal assessment (14–16). Maternal adverse events included eclampsia, placental abruption, pulmonary edema, acute kidney injury, disseminated intravascular coagulation, and intensive care unit admission. Fetal or neonatal adverse events included fetal distress requiring urgent delivery, intrauterine fetal death or stillbirth, neonatal compromised condition requiring immediate resuscitation, and neonatal death. Neonatal compromised condition requiring immediate resuscitation was defined as a 5-min Apgar score <7 and/or the need for immediate neonatal resuscitation documented by the attending neonatologist. Acute kidney injury was defined as newly developed renal dysfunction documented during hospitalization, based on increased serum creatinine levels and clinical diagnosis by the treating obstetric team. Placental abruption, pulmonary edema, disseminated intravascular coagulation, fetal distress, stillbirth, and neonatal death were diagnosed according to standard obstetric and neonatal clinical criteria and were recorded in the electronic medical records.

To avoid potential overlap between predictors and outcomes, fetal growth restriction, oligohydramnios, and HELLP syndrome identified at admission or during the initial clinical evaluation were treated as baseline pregnancy-related complications or candidate predictors and were not counted as components of the composite adverse pregnancy outcome. The detailed definitions and classification of all outcome components are provided in Supplementary Table S1. Candidate predictor variables were selected based on clinical relevance, previous literature, and routine availability at the time of admission-based assessment after EOPE diagnosis. To ensure temporal alignment and reduce the risk of circularity, all candidate predictors were defined as variables measured or diagnosed at admission or within the first 24 h after admission, before the occurrence of the predefined composite adverse pregnancy outcome. The full list of candidate variables initially entered into the LASSO regression model included maternal age, pre-pregnancy body mass index, parity, chronic hypertension, pregestational diabetes, gestational age at admission, systolic blood pressure, diastolic blood pressure, urine protein score, platelet count, AST, ALT, serum creatinine, uric acid, lactate dehydrogenase, hemoglobin, fetal growth restriction, oligohydramnios, and HELLP syndrome. Fetal growth restriction, oligohydramnios, and HELLP syndrome were considered baseline pregnancy-related complications because they are clinically relevant to EOPE severity, placental dysfunction, fetal compromise, or maternal organ involvement and can usually be identified at the time of initial admission assessment using routine obstetric evaluation, fetal ultrasound assessment, laboratory testing, and medical record review. These variables were treated as candidate predictors only if they were documented at admission or during the initial clinical evaluation within 24 h after admission, and they were not counted as components of the composite adverse pregnancy outcome. Other pregnancy-, delivery-, treatment-, or neonatal-related variables, including delivery mode, gestational age at delivery, neonatal birth weight, neonatal intensive care unit admission, magnesium sulfate administration, and components of the composite adverse pregnancy outcome, were excluded *a priori* because they occurred after the prediction time point, reflected subsequent clinical management, or represented outcome-related information. This approach was used to preserve the temporal sequence between predictors and outcomes and to reduce the risk of circularity. First-trimester aspirin prophylaxis and medication history for autoimmune diseases before or during early pregnancy were considered potentially relevant; however, these variables were not consistently documented in the retrospective inpatient records and, therefore, were not included as candidate predictors to avoid substantial missingness and information bias. The complete list of variables considered for model development and the rationale for inclusion or exclusion are provided in Supplementary Table S4.

### Data sources

All data were obtained from the hospital electronic medical record system. Demographic and obstetric information was extracted from admission records and obstetric databases. Blood pressure measurements were performed by trained clinical staff using standard hospital protocols at the time of admission. Laboratory parameters were measured using automated analyzers in the hospital’s central laboratory following standardized quality control procedures. Baseline pregnancy-related complications, including fetal growth restriction, oligohydramnios, and HELLP syndrome, were considered candidate predictors only when they were diagnosed at admission or during the initial clinical evaluation within 24 h after admission according to established clinical criteria and documented by attending obstetricians. These complications were selected because they were routinely evaluated in EOPE patients at admission and were consistently documented in the electronic medical records. Events or complications newly developing after this initial assessment were not used as predictors.

### Bias

Several measures were implemented to minimize potential sources of bias. First, consecutive patients meeting the eligibility criteria during the study period were included to reduce selection bias. Second, all variables were obtained from routinely recorded clinical data to limit information bias. Third, the study used predefined inclusion and exclusion criteria to ensure consistency in participant selection. Finally, internal validation using a randomly divided validation cohort was performed to evaluate the stability and robustness of the prediction model.

### Study size

The sample size was determined based on the number of eligible patients available during the study period. A total of 425 patients with early-onset preeclampsia were included. The sample size was considered adequate for predictive modeling because the number of outcome events allowed sufficient events per variable (EPV) for reliable model development. The dataset was randomly divided into a training cohort (70%) and a validation cohort (30%) to enable model development and internal validation.

### Quantitative variables

Continuous variables were analyzed as quantitative variables and summarized as means with standard deviations. Variables such as gestational age at admission, systolic blood pressure, platelet count, AST level, and uric acid level were treated as continuous predictors in the regression analyses. To improve clinical interpretability, odds ratios for continuous predictors were reported using clinically meaningful increments when appropriate: per 1-week increase for gestational age at admission, per 10-mmHg increase for systolic blood pressure, per 1-grade increase for urine protein score, per 50 × 10^9^/L decrease for platelet count, per 10-U/L increase for AST, and per 50-μmol/L increase for uric acid. Categorical variables were presented as counts and percentages. For baseline comparison, urine protein score was additionally reclassified as 0–1 + versus ≥2 + to improve clinical interpretability, whereas the original ordinal score was retained in regression modeling to preserve information.

### Statistical methods

All statistical analyses were performed using R software (version 4.3.1; R Foundation for Statistical Computing, Vienna, Austria). The distribution of continuous variables was assessed using the Shapiro–Wilk test and visual inspection of histograms or quantile–quantile plots. Normally distributed continuous variables were presented as mean ± standard deviation and compared using the Student’s t-test. Non-normally distributed continuous variables were presented as median with interquartile range and compared using the Mann–Whitney U test. Categorical variables were summarized as counts (percentages) and compared using the chi-square test or Fisher’s exact test, as appropriate. Feature screening was conducted in the training cohort using least absolute shrinkage and selection operator (LASSO) regression with 10-fold cross-validation to reduce the dimensionality of candidate predictors and identify variables with stable predictive signals. Variables with non-zero coefficients under the one-standard-error rule (*λ*₁se) were recorded as LASSO-selected variables. To avoid excluding clinically established predictors that may be important for EOPE risk assessment, variables selected by LASSO, together with variables showing statistical significance in univariable logistic regression and clear clinical relevance, were considered for multivariable logistic regression. The final prediction model was determined based on independent associations in the multivariable logistic regression model, clinical interpretability, and model parsimony. A nomogram was constructed based on the final multivariable model to provide an individualized risk estimation tool. The performance of the prediction model was evaluated in terms of discrimination, calibration, and clinical utility. Discrimination was assessed using the area under the receiver operating characteristic curve (AUC). Calibration was evaluated using calibration curves comparing predicted and observed probabilities. Clinical usefulness was assessed using decision curve analysis (DCA) to estimate the net benefit across a range of threshold probabilities. To examine the robustness of the model and address potential circularity related to baseline pregnancy-related complications, a sensitivity analysis was performed by excluding fetal growth restriction and HELLP syndrome from the final prediction model. The sensitivity model was refitted using the remaining admission-based clinical and laboratory predictors, and its discrimination, calibration, and clinical utility were evaluated in both the training and validation cohorts using the same procedures as those applied to the primary model. Missing data were assessed for each candidate predictor and the composite adverse pregnancy outcome. Because no missing values were present in the final analytic dataset, complete-case analysis was performed using all 425 eligible patients, and multiple imputation was not required. The missing-data proportions for all variables are presented in Supplementary Table S3. Internal validation was primarily performed using the split-sample validation cohort to assess the model’s discrimination, calibration, and clinical utility. Ten-fold cross-validation was used only within the LASSO regression procedure to select the optimal penalty parameter and identify variables with stable predictive signals; it was not used as a separate validation procedure for the final prediction model. Bootstrap resampling with 1,000 repetitions was used to generate the bias-corrected calibration curve in the training cohort and to visually assess potential calibration optimism. However, full bootstrap-based optimism correction for all performance measures, including AUC, calibration slope, and decision curve analysis, was not performed. A two-sided *p*-value < 0.05 was considered statistically significant.

## Results

### Baseline characteristics of the study population

The baseline demographic, obstetric, and laboratory characteristics of the study population are summarized in Table 1, including the overall cohort (*n* = 425), the training cohort (*n* = 298), and the validation cohort (*n* = 127). No missing values were observed for any candidate predictor or the composite adverse pregnancy outcome in the final analytic dataset, and all 425 patients were included in the complete-case analysis. Overall, the distributions of most clinical variables were comparable between the training and validation cohorts, indicating adequate balance between the two datasets. The mean maternal age of the participants was 31.03 ± 5.02 years, and the mean pre-pregnancy body mass index (BMI) was 24.08 ± 4.02 kg/m^2^. Approximately half of the women were primiparous (50.6%), while 17.4% had chronic hypertension and 5.9% had pregestational diabetes. At admission, the mean gestational age was 29.00 ± 2.31 weeks, with mean systolic and diastolic blood pressures of 167.35 ± 17.11 mmHg and 106.61 ± 11.06 mmHg, respectively. Regarding laboratory findings, the mean platelet count was 188.80 ± 53.92 × 10^9^/L, AST level 48.85 ± 23.19 U/L, ALT level 50.49 ± 23.90 U/L, serum creatinine 68.80 ± 16.96 μmol/L, uric acid 425.19 ± 86.95 μmol/L, LDH 338.34 ± 122.70 U/L, and hemoglobin 110.90 ± 12.54 g/L. Obstetric complications were common in this population, with fetal growth restriction observed in 65.9% of cases, oligohydramnios in 47.1%, and HELLP syndrome in 42.4%. Magnesium sulfate was administered to the majority of patients (79.3%). Importantly, no significant differences were observed between the training and validation cohorts for most baseline variables (all *p* > 0.05). After reclassification of urine protein score into 0–1 + and ≥2+, its distribution remained significantly different between the training and validation cohorts (*p* = 0.003). Specifically, the proportion of patients with urine protein ≥2 + was higher in the training cohort than in the validation cohort (80.2% vs. 66.1%). The proportion of the predefined composite adverse pregnancy outcome was also comparable between the training and validation cohorts (34.9% vs. 38.6%, *p* = 0.539).

**Table 1:** Baseline characteristics of patients with early-onset preeclampsia in the training and validation cohorts.

Variable	Overall cohort (*n* = 425)	Training cohort (*n* = 298)	Validation cohort (*n* = 127)	*P-*value*
Maternal age, years	31.03 (5.02)	30.99 (5.09)	31.14 (4.85)	0.775
Pre-pregnancy BMI, kg/m^2^	24.08 (4.02)	24.14 (4.05)	23.93 (3.96)	0.623
Primipara, *n* (%)				0.633
No	210 (49.4)	150 (50.3)	60 (47.2)	
Yes	215 (50.6)	148 (49.7)	67 (52.8)	
Chronic hypertension, *n* (%)				0.652
No	351 (82.6)	244 (81.9)	107 (84.3)	
Yes	74 (17.4)	54 (18.1)	20 (15.7)	
Pregestational diabetes, *n* (%)				0.361
No	400 (94.1)	283 (95.0)	117 (92.1)	
Yes	25 (5.9)	15 (5.0)	10 (7.9)	
Gestational age at admission, weeks	29.00 (2.31)	29.05 (2.28)	28.88 (2.40)	0.482
Systolic blood pressure, mmHg	167.35 (17.11)	167.71 (17.01)	166.50 (17.38)	0.507
Diastolic blood pressure, mmHg	106.61 (11.06)	106.52 (11.00)	106.82 (11.24)	0.797
Urine protein category, *n* (%)				0.003
0–1+	102 (24.0)	59 (19.8)	43 (33.9)	
≥2+	323 (76.0)	239 (80.2)	84 (66.1)	
Platelet count, ×10^9^/L	188.80 (53.92)	188.91 (56.10)	188.56 (48.64)	0.952
AST, U/L	48.85 (23.19)	48.56 (22.83)	49.54 (24.10)	0.692
ALT, U/L	50.49 (23.90)	50.82 (24.51)	49.72 (22.47)	0.666
Serum creatinine, μmol/L	68.80 (16.96)	68.34 (17.22)	69.89 (16.37)	0.388
Uric acid, μmol/L	425.19 (86.95)	424.70 (85.99)	426.32 (89.50)	0.861
LDH, U/L	338.34 (122.70)	340.20 (118.53)	333.96 (132.38)	0.632
Hemoglobin, g/L	110.90 (12.54)	111.20 (12.71)	110.19 (12.16)	0.445
Fetal growth restriction, *n* (%)				0.794
No	145 (34.1)	100 (33.6)	45 (35.4)	
Yes	280 (65.9)	198 (66.4)	82 (64.6)	
Oligohydramnios, *n* (%)				0.955
No	225 (52.9)	157 (52.7)	68 (53.5)	
Yes	200 (47.1)	141 (47.3)	59 (46.5)	
HELLP syndrome, *n* (%)				0.879
No	245 (57.6)	173 (58.1)	72 (56.7)	
Yes	180 (42.4)	125 (41.9)	55 (43.3)	
Magnesium sulfate administration, *n* (%)				0.402
No	88 (20.7)	58 (19.5)	30 (23.6)	
Yes	337 (79.3)	240 (80.5)	97 (76.4)	
Adverse pregnancy outcome (composite), *n* (%)				0.539
No	272 (64.0)	194 (65.1)	78 (61.4)	
Yes	153 (36.0)	104 (34.9)	49 (38.6)	

Data are presented as mean (standard deviation) for continuous variables and number (percentage) for categorical variables. Urine protein score was reclassified as 0–1 + versus ≥2 + for baseline comparison, whereas the original ordinal score was used in regression modeling to preserve information. BMI, body mass index; SBP, systolic blood pressure; DBP, diastolic blood pressure; AST, aspartate aminotransferase; ALT, alanine aminotransferase; LDH, lactate dehydrogenase; FGR, fetal growth restriction; HELLP, hemolysis, elevated liver enzymes, and low platelet syndrome. The composite adverse pregnancy outcome was defined as the occurrence of at least one predefined maternal or fetal/neonatal adverse event after admission, as detailed in Supplementary Table S1. Baseline fetal growth restriction, oligohydramnios, and HELLP syndrome were not included as components of the composite outcome. No missing values were observed for any variable included in the final analytic dataset.

### Feature selection using LASSO regression

**Figure 1:**
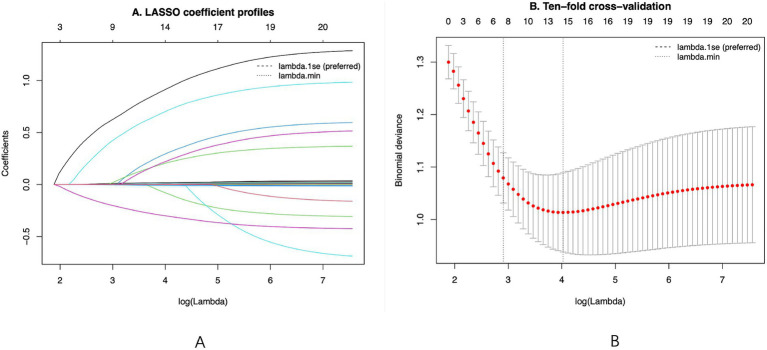
Feature selection using the least absolute shrinkage and selection operator (LASSO) regression. **(A)** LASSO coefficient profiles of the candidate predictors plotted against the logarithm of the penalty parameter (log *λ*). Each colored line represents the trajectory of a regression coefficient as λ changes. With increasing penalization, the coefficients gradually shrink toward zero, allowing identification of the most relevant predictors. **(B)** Ten-fold cross-validation within the LASSO procedure for selecting the optimal penalty parameter (λ). The red dots represent the mean binomial deviance, and the vertical bars indicate ±1 standard error. The two dashed vertical lines correspond to the optimal values of λ determined by the minimum criterion (λ_min) and the one-standard-error criterion (λ_1se). Variables with non-zero coefficients at λ_1se were selected as candidate predictors for subsequent multivariable logistic regression analysis.

Feature selection was performed using the least absolute shrinkage and selection operator (LASSO) regression, and the results are presented in Figure 1 and Table 2. Ten-fold cross-validation was applied to determine the optimal penalty parameter (*λ*). As shown in Figure 1A, the regression coefficients of candidate predictors gradually shrank toward zero as the value of λ increased. The optimal λ was selected according to the one-standard-error criterion (λ₁se), which resulted in a more parsimonious model while maintaining stable predictive performance (Figure 1B). Using this criterion, seven variables with non-zero coefficients were identified as LASSO-selected variables, including HELLP syndrome, fetal growth restriction, gestational age at admission, systolic blood pressure, platelet count, uric acid, and AST (Table 2). These variables were subsequently considered together with clinically relevant variables that were significantly associated with the outcome in univariable analysis, including urine protein score, chronic hypertension, and oligohydramnios, in the multivariable logistic regression analysis. Among these predictors, HELLP syndrome (coefficient = 0.597) and fetal growth restriction (coefficient = 0.388) exhibited relatively larger positive coefficients, indicating stronger associations with adverse pregnancy outcomes, whereas gestational age at admission (coefficient = −0.191) and platelet count (coefficient = −0.003) showed negative coefficients, suggesting potential protective effects.

**Table 2:** Variables with non-zero coefficients in LASSO regression in the training cohort.

Variable	Coefficient
HELLP syndrome (Yes vs. No)	0.597
Fetal growth restriction (Yes vs. No)	0.388
Gestational age at admission (weeks)	−0.191
Systolic blood pressure (mmHg)	0.009
Platelet count (×10^9^/L)	−0.003
Uric acid (μmol/L)	0.002
AST (U/L)	0.002

LASSO regression with ten-fold cross-validation was used for initial variable screening and dimensionality reduction in the training cohort. The tuning parameter (λ) was selected according to the one-standard-error rule (λ₁se) to obtain a parsimonious set of variables with stable predictive signals. Variables with non-zero coefficients at λ₁se were recorded as LASSO-selected variables. These variables, together with clinically relevant variables showing significant associations in univariable analysis, were considered for subsequent multivariable logistic regression analysis. The final prediction model was determined based on independent associations in the multivariable model, clinical interpretability, and model parsimony. The full list of variables initially entered into the LASSO model is provided in Supplementary Table S4. AST, aspartate aminotransferase; HELLP, hemolysis, elevated liver enzymes, and low platelet syndrome; LASSO, least absolute shrinkage and selection operator.

### Univariate and multivariable logistic regression analyses

**Figure 2:**
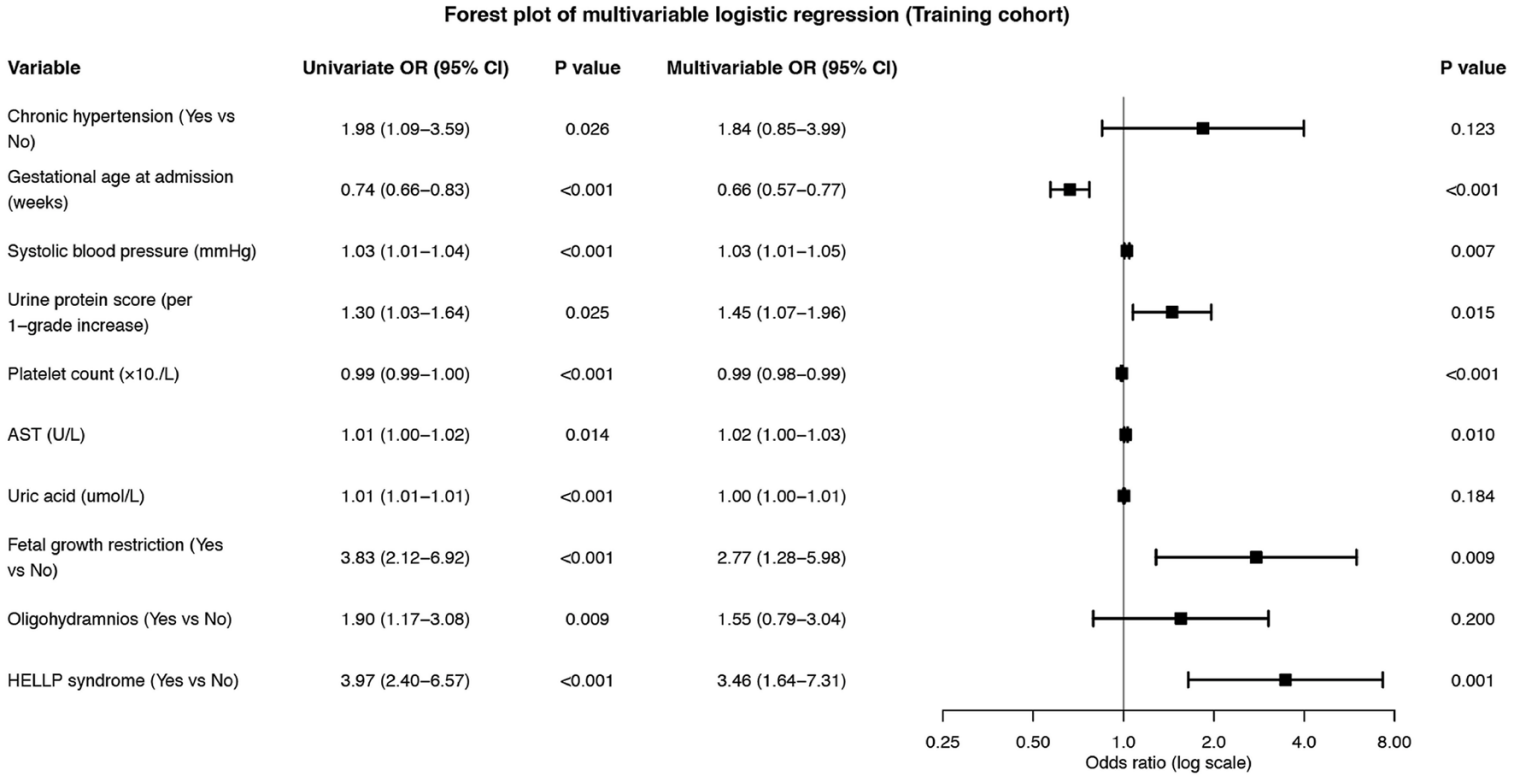
Forest plot of univariate and multivariable logistic regression analyses for predictors of adverse pregnancy outcomes in the training cohort. The forest plot illustrates the odds ratios (ORs) and 95% confidence intervals (CIs) for candidate predictors of adverse pregnancy outcomes. The vertical reference line represents an OR of 1. Variables located to the right of the line indicate an increased risk of adverse outcomes, whereas those to the left indicate a protective effect. Results from univariate logistic regression are presented in the table for reference, while the forest plot displays the adjusted ORs from the multivariable logistic regression model. Independent predictors identified in the multivariable analysis were subsequently incorporated into the prediction nomogram.

The associations between clinical variables and adverse pregnancy outcomes were evaluated using univariate and multivariable logistic regression analyses in the training cohort, as presented in Table 3 and illustrated in Figure 2. In the univariate analysis, several variables were significantly associated with adverse pregnancy outcomes, including chronic hypertension, gestational age at admission, systolic blood pressure, urine protein score, platelet count, AST, uric acid, fetal growth restriction, oligohydramnios, and HELLP syndrome (all *p* < 0.05). After adjustment in the multivariable logistic regression model, seven variables remained independently associated with adverse pregnancy outcomes. In clinically interpretable units, each 1-week increase in gestational age at admission was associated with a lower risk of adverse pregnancy outcomes (OR = 0.67, 95% CI: 0.58–0.77, *p* < 0.001), whereas each 10-mmHg increase in systolic blood pressure was associated with a higher risk (OR = 1.35, 95% CI: 1.13–1.62, *p* = 0.001). Each 1-grade increase in urine protein score was also associated with increased risk (OR = 1.43, 95% CI: 1.06–1.92, *p* = 0.018). For platelet count, each 50 × 10^9^/L decrease was associated with increased risk (OR = 1.85, 95% CI: 1.39–2.47, *p* < 0.001). Each 10-U/L increase in AST was associated with increased risk (OR = 1.20, 95% CI: 1.06–1.37, *p* = 0.006). Fetal growth restriction (OR = 3.56, 95% CI: 1.76–7.17, *p* < 0.001) and HELLP syndrome (OR = 4.57, 95% CI: 2.45–8.53, *p* < 0.001) were also independently associated with adverse pregnancy outcomes. In contrast, chronic hypertension, uric acid levels, and oligohydramnios were not independently associated with adverse pregnancy outcomes after adjustment (*p* > 0.05). The magnitude and direction of these associations are visualized in the forest plot (Figure 2).

**Table 3:** Univariate and multivariable logistic regression analyses of risk factors for adverse pregnancy outcomes in the training cohort.

Variable	Univariate OR (95% CI)	*P*-value	Multivariable OR (95% CI)	*P*-value
Chronic hypertension, yes *vs* no	1.98 (1.09–3.59)	0.026	1.84 (0.85–3.99)	0.123
Gestational age at admission, per 1-week increase	0.74 (0.66–0.83)	<0.001	0.67 (0.58–0.77)	<0.001
Systolic blood pressure, per 10-mmHg increase	1.30 (1.13–1.48)	<0.001	1.35 (1.13–1.62)	0.001
Urine protein score, per 1-grade increase	1.30 (1.03–1.64)	0.025	1.43 (1.06–1.92)	0.018
Platelet count, per 50 × 10^9^/L decrease	1.64 (1.28–2.11)	<0.001	1.85 (1.39–2.47)	<0.001
AST, per 10-U/L increase	1.10 (1.02–1.19)	0.014	1.20 (1.06–1.37)	0.006
Uric acid, per 50-μmol/L increase	1.43 (1.24–1.64)	<0.001	1.11 (0.95–1.30)	0.184
Fetal growth restriction, yes *vs* no	3.83 (2.12–6.92)	<0.001	3.56 (1.76–7.17)	<0.001
Oligohydramnios, yes *vs* no	1.90 (1.17–3.08)	0.009	1.55 (0.79–3.04)	0.200
HELLP syndrome, yes *vs* no	3.97 (2.40–6.57)	<0.001	4.57 (2.45–8.53)	<0.001

Data are presented as odds ratios (ORs) with 95% confidence intervals (CIs). *p*-values were obtained from logistic regression models. For continuous predictors, ORs are presented using clinically interpretable increments: per 1-week increase for gestational age at admission, per 10-mmHg increase for systolic blood pressure, per 1-grade increase for urine protein score, per 50 × 10^9^/L decrease for platelet count, per 10-U/L increase for AST, and per 50-μmol/L increase for uric acid. For categorical predictors, ORs compare “Yes” versus “No” as indicated. AST, aspartate aminotransferase; CI, confidence interval; HELLP, hemolysis, elevated liver enzymes, and low platelet syndrome; OR, odds ratio.

### Nomogram construction

**Figure 3:**
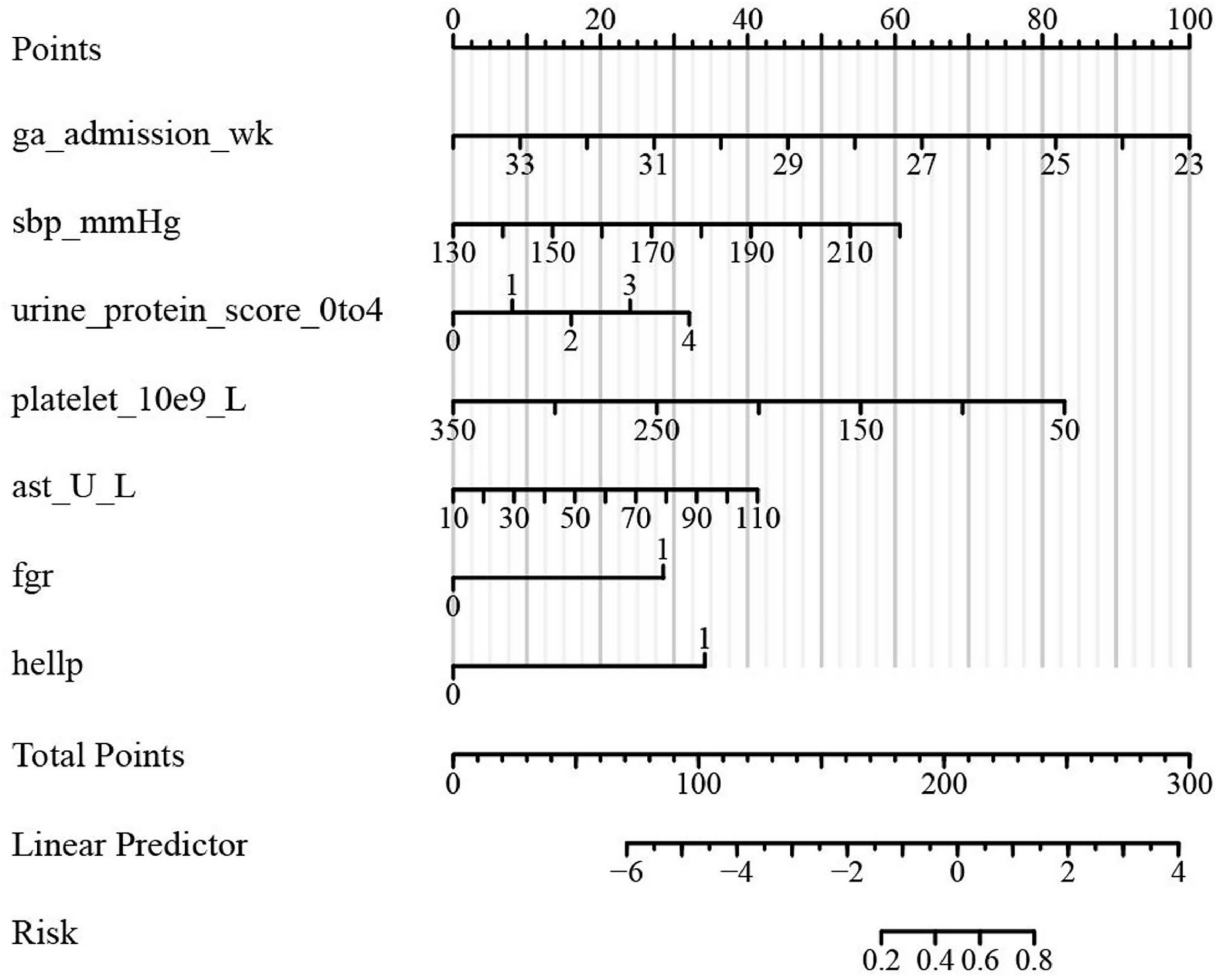
Nomogram for predicting the probability of the composite adverse pregnancy outcome in women with early-onset preeclampsia (EOPE). The nomogram was developed based on the final multivariable logistic regression model. Each predictor corresponds to a specific point value on the top scale. The points assigned to all predictors are summed to obtain a total score, which is then mapped to the predicted probability of the composite adverse pregnancy outcome on the bottom scale. For clinical interpretation, predicted risks were categorized exploratorily as low risk (<20%), intermediate risk (20–50%), and high risk (>50%). These thresholds have not been externally validated and are intended only to facilitate preliminary risk stratification and clinical communication. They should not be used as definitive clinical decision cutoffs or as stand-alone criteria for monitoring intensity, intervention, expectant management, or timing of delivery. The predictors included in the nomogram were gestational age at admission, systolic blood pressure, urine protein score, platelet count, AST level, fetal growth restriction (FGR), and HELLP syndrome.

Based on the independent predictors identified in the multivariable logistic regression analysis, a nomogram was developed to estimate the individualized risk of the composite adverse pregnancy outcome (Figure 3). The nomogram incorporated seven predictors: gestational age at admission, systolic blood pressure, urine protein score, platelet count, AST level, fetal growth restriction, and HELLP syndrome. Each predictor was assigned a specific score according to its regression coefficient. The total score, calculated by summing the points corresponding to each predictor, was then mapped to the predicted probability of the composite adverse pregnancy outcome. To illustrate the clinical use of the nomogram, we provide a representative example. For a woman with EOPE admitted at 28 weeks of gestation, with systolic blood pressure of 180 mmHg, urine protein score of 3+, platelet count of 120 × 10^9^/L, AST level of 80 U/L, fetal growth restriction, and no HELLP syndrome, the estimated predicted probability of the composite adverse pregnancy outcome was approximately 78% based on the final model. This patient would therefore be classified as high risk, suggesting the need for intensified maternal-fetal monitoring, multidisciplinary assessment, and individualized discussion regarding the timing of delivery. For clinical interpretation, we proposed exploratory risk categories based on predicted probabilities: low risk (<20%), intermediate risk (20–50%), and high risk (>50%). These thresholds were intended only to facilitate preliminary risk stratification and clinical communication. They have not been externally validated and should not be used as definitive clinical decision cutoffs or as stand-alone criteria for determining monitoring intensity, intervention, expectant management, or timing of delivery.

### Model performance in the training cohort

**Figure 4:**
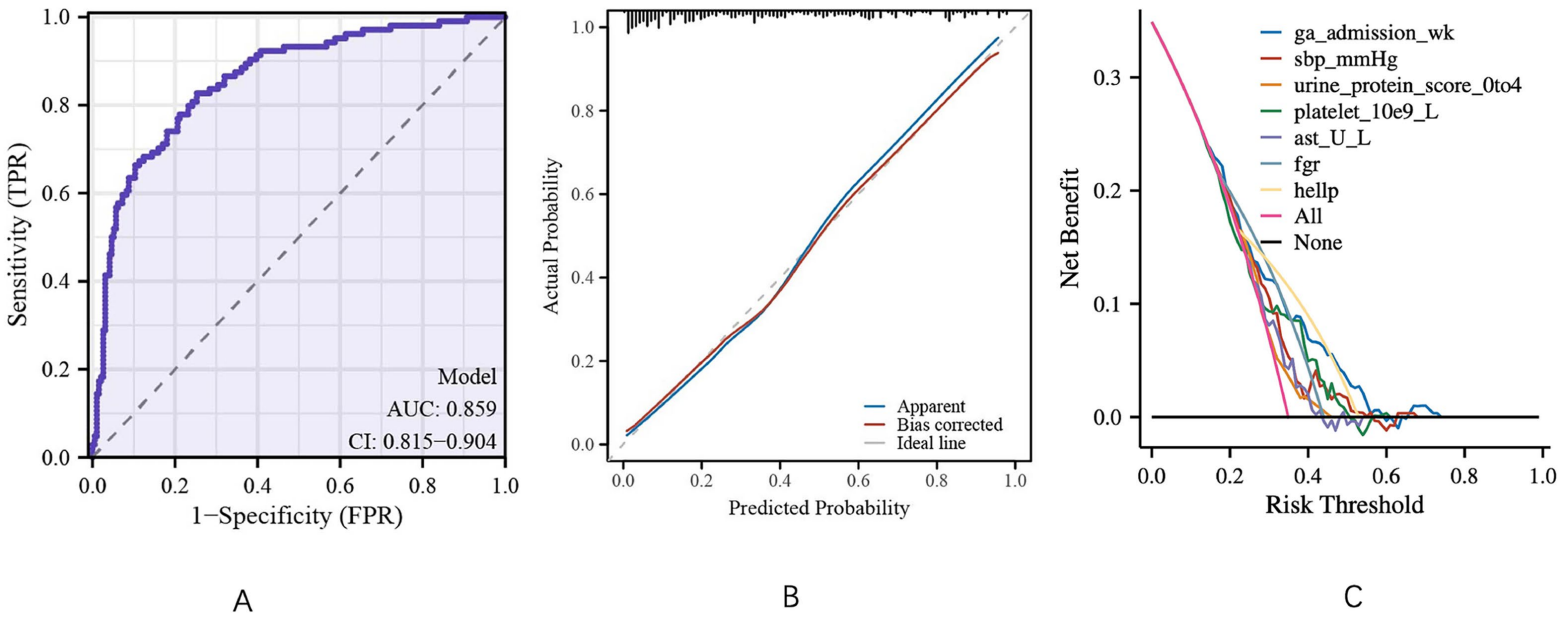
Performance of the prediction model in the training cohort. **(A)** Receiver operating characteristic (ROC) curve showing the discriminative ability of the model, with an area under the curve (AUC) of 0.859 (95% CI, 0.815–0.904). **(B)** Calibration curve assessing the agreement between predicted probabilities and observed outcomes. The apparent curve represents calibration performance in the training dataset, the bias-corrected curve was generated using bootstrap resampling to visually assess calibration optimism, and the diagonal dashed line indicates perfect calibration. **(C)** Decision curve analysis (DCA) evaluates the clinical utility of the prediction model across threshold probabilities. In the training cohort, the model provided greater net benefit than the “treat-all” and “treat-none” strategies across threshold probabilities of approximately 10–80%.

The predictive performance of the model in the training cohort was evaluated in terms of discrimination, calibration, and clinical utility (Figure 4). The receiver operating characteristic (ROC) curve demonstrated good discriminative ability, with an area under the curve (AUC) of 0.859 (95% CI: 0.815–0.904) (Figure 4A). The apparent and bootstrap bias-corrected calibration curves showed good agreement between the predicted and observed probabilities, indicating satisfactory model calibration in the training cohort (Figure 4B). Decision curve analysis further demonstrated that the prediction model provided greater net clinical benefit than both the “treat-all” and “treat-none” strategies across threshold probabilities of approximately 10–80% in the training cohort, suggesting potential clinical usefulness for risk-stratified management within this range (Figure 4C).

### Model validation in the validation cohort

**Figure 5:**
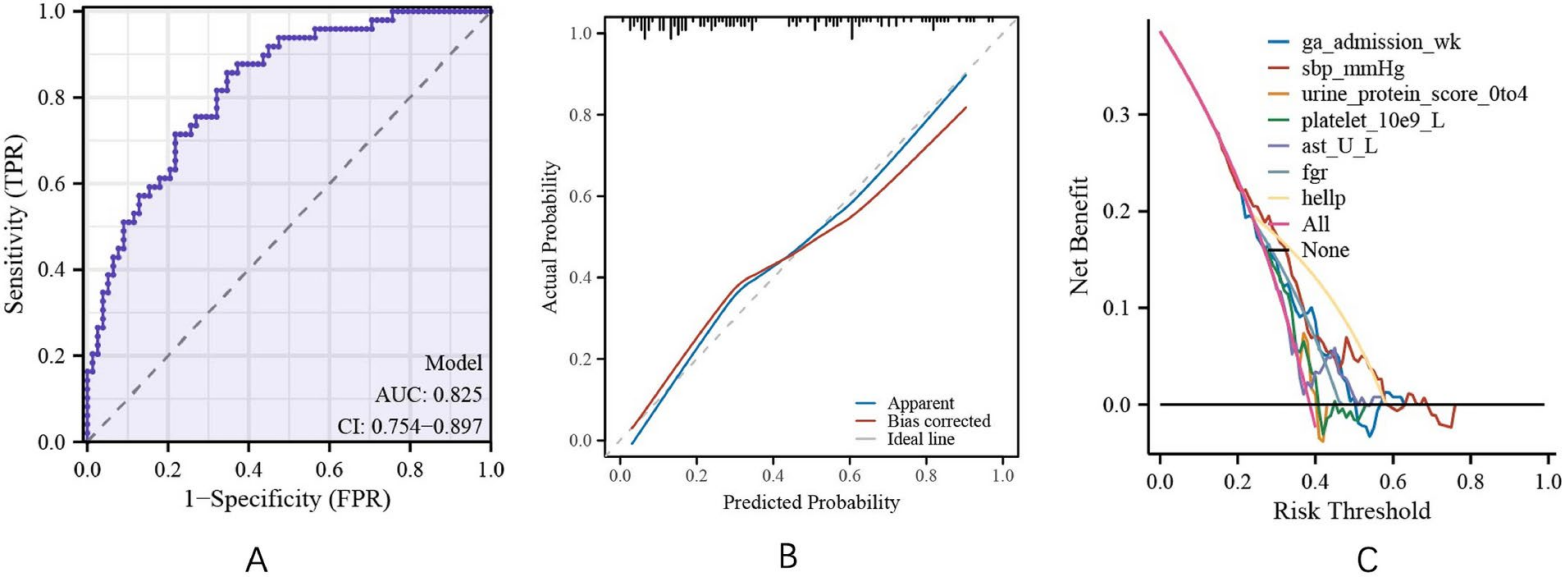
Validation of the prediction model in the independent validation cohort. **(A)** Receiver operating characteristic (ROC) curve demonstrating the discriminative performance of the model in the validation cohort, with an AUC of 0.825 (95% CI, 0.754–0.897). **(B)** Calibration curve showing the agreement between predicted and observed probabilities of adverse pregnancy outcomes in the validation dataset. **(C)** Decision curve analysis (DCA) evaluates the clinical usefulness of the prediction model in the validation cohort. The model provided greater net benefit than the “treat-all” and “treat-none” strategies across threshold probabilities of approximately 15–70%.

The predictive model was subsequently evaluated in the split-sample validation cohort to assess its internal validity and robustness (Figure 5). The ROC analysis indicated that the model maintained good discriminative performance in the validation dataset, with an AUC of 0.825 (95% CI: 0.754–0.897) (Figure 5A). Calibration analysis demonstrated satisfactory agreement between predicted and observed risks (Figure 5B). Decision curve analysis also showed that the model provided greater net benefit than the “treat-all” and “treat-none” strategies across threshold probabilities of approximately 15–70% in the validation cohort (Figure 5C), supporting the potential clinical utility of the proposed prediction model for risk-stratified monitoring and individualized management.

### Sensitivity analysis excluding fetal growth restriction and HELLP syndrome

To further assess the robustness of the prediction model and address potential concerns regarding circularity, we performed a sensitivity analysis by excluding fetal growth restriction and HELLP syndrome from the model. The sensitivity model was refitted using the remaining admission-based predictors, including gestational age at admission, systolic blood pressure, urine protein score, platelet count, and AST level. The sensitivity model retained moderate discriminative performance, with an AUC of 0.778 (95% CI: 0.723–0.832) in the training cohort and 0.722 (95% CI: 0.630–0.809) in the validation cohort. Calibration was acceptable in the training cohort, with a Hosmer–Lemeshow χ^2^ of 7.23 and a *p*-value of 0.513, whereas calibration in the validation cohort was less satisfactory, with a Hosmer–Lemeshow χ^2^ of 17.98 and a *p*-value of 0.021. These findings suggest that the model retained discriminatory ability after excluding fetal growth restriction and HELLP syndrome, although its performance was attenuated compared with the primary model. The performance of the sensitivity model is summarized in Supplementary Table S2.

## Discussion

Early-onset preeclampsia (EOPE) is widely recognized as one of the most severe hypertensive disorders of pregnancy and is associated with substantial maternal and fetal morbidity. In this study, we developed and internally validated a nomogram-based prediction model for adverse pregnancy outcomes among women already diagnosed with EOPE at admission, using routinely available clinical variables. The model incorporated seven admission-based predictors—gestational age at admission, systolic blood pressure, urine protein score, platelet count, AST level, fetal growth restriction, and HELLP syndrome—and demonstrated good predictive performance, with an AUC of 0.859 in the training cohort and 0.825 in the validation cohort. These findings suggest that the proposed model may serve as a preliminary tool for admission-based risk stratification and individualized clinical assessment after EOPE diagnosis, pending external validation. Importantly, this model was not designed for early prenatal prediction, population-based screening, or prediction of the initial occurrence of EOPE.

Several predictors identified in our model are consistent with the current understanding of the pathophysiology and clinical progression of EOPE. Gestational age at admission was an important protective factor, with lower gestational age significantly associated with an increased risk of adverse pregnancy outcomes. This finding aligns with previous studies indicating that earlier disease onset often reflects more severe placental dysfunction and systemic endothelial injury, which are strongly associated with poor pregnancy outcomes (17, 18). Similarly, elevated systolic blood pressure was independently associated with adverse outcomes in our study. Severe hypertension has long been recognized as a major indicator of disease severity in preeclampsia and is closely linked to maternal complications such as eclampsia, placental abruption, and organ dysfunction (19–21).

Laboratory parameters also played an important role in predicting adverse outcomes. Platelet count and AST levels were independently associated with the risk of adverse pregnancy outcomes in the present study. Thrombocytopenia is commonly observed in severe preeclampsia and may reflect platelet consumption and endothelial activation associated with systemic inflammation and microvascular injury (22). Elevated AST levels indicate hepatic involvement and are often observed in severe forms of the disease, including HELLP syndrome (23). Consistent with previous research, HELLP syndrome was identified as one of the strongest predictors in our model, highlighting its well-established association with severe maternal and perinatal complications (24, 25).

In addition to conventional laboratory indicators, emerging evidence suggests that additional biochemical markers may further improve risk assessment in preeclampsia. Blood lipid parameters have been increasingly investigated because dyslipidemia may be related to endothelial dysfunction, oxidative stress, abnormal placentation, and metabolic disturbances in preeclampsia. A recent study reported that renal function markers combined with blood lipid levels may have clinical value for predicting preeclampsia (26). These findings suggest that lipid-related indicators may provide complementary information beyond conventional clinical and laboratory variables. However, in the present retrospective cohort, blood lipid parameters were not routinely measured in all patients with EOPE at admission; therefore, incorporating them into the prediction model would have introduced substantial missing data and potential selection bias. Future prospective studies should systematically collect lipid profiles and evaluate whether they improve model discrimination, calibration, and clinical utility for predicting adverse pregnancy outcomes in EOPE.

In addition, fetal growth restriction (FGR) emerged as an important predictor of adverse pregnancy outcomes. FGR is frequently associated with placental insufficiency and impaired uteroplacental perfusion, which are central features in the pathogenesis of EOPE (27, 28). Previous studies have shown that the coexistence of EOPE and FGR substantially increases the risk of preterm delivery, fetal distress, and perinatal mortality (29–31). The inclusion of FGR in our model, therefore, reflects the close relationship between placental dysfunction and poor pregnancy outcomes.

A notable strength of the present study is the development of a clinically applicable prediction model based on routinely available clinical and laboratory variables. Compared with models relying on specialized biomarkers or advanced imaging techniques, our model can be easily implemented in routine obstetric practice. It is also important to interpret our findings in the context of existing prediction tools for preeclampsia-related adverse outcomes. The fullPIERS model is one of the most established tools for predicting adverse maternal outcomes in women with preeclampsia and includes predictors such as gestational age, chest pain or dyspnea, oxygen saturation, platelet count, serum creatinine, and AST (32). The miniPIERS model was developed to support risk assessment and triage in low-resource settings and uses a simpler set of clinical predictors (33). The PREP-L and PREP-S models were specifically developed for women with early-onset preeclampsia and provide individualized estimates of adverse maternal outcome risk by discharge or at different time points after diagnosis (34). Compared with these models, our nomogram has several distinct features. First, our model was developed in a Chinese EOPE cohort from a tertiary referral center, which may better reflect the clinical characteristics and management context of this population. Second, whereas fullPIERS, miniPIERS, and PREP primarily focus on adverse maternal outcomes, our model was designed to predict a composite adverse pregnancy outcome, including both maternal and fetal/neonatal events. Third, our model incorporated routinely available admission-based variables, including urine protein score and fetal growth restriction, which may improve its relevance to obstetric risk stratification in EOPE. However, unlike fullPIERS and PREP, our model has not yet undergone external validation, and its applicability outside the present setting remains to be established. Therefore, our nomogram should be viewed as a complementary and internally validated risk-stratification tool rather than a replacement for established prediction models. A structured comparison between the present nomogram and existing prediction tools is provided in Supplementary Table S5.

An important methodological consideration in prediction modeling is the temporal relationship between predictors and outcomes. In the present study, all predictors were restricted to variables available at admission or within the first 24 h after admission, before the occurrence of the predefined composite adverse pregnancy outcome. Fetal growth restriction, oligohydramnios, and HELLP syndrome were considered as candidate predictors only when diagnosed at admission or during the initial clinical evaluation, because these baseline pregnancy-related complications were routinely assessed in EOPE patients and reflected placental dysfunction, fetal compromise, or maternal organ involvement. They were therefore treated as predictors rather than outcome components. To further address the possibility of circularity, we performed a sensitivity analysis excluding fetal growth restriction and HELLP syndrome. The sensitivity model retained moderate discriminative ability, although its performance was attenuated compared with the primary model. Nevertheless, this issue highlights the importance of careful temporal definition in future prospective validation studies. In the intended admission-based setting, the nomogram provides an intuitive tool that allows clinicians to estimate the individualized risk of adverse pregnancy outcomes among patients already diagnosed with EOPE and may support clinical communication regarding monitoring intensity, timing of delivery, and multidisciplinary management. The decision curve analysis further supports the potential clinical usefulness of the nomogram by demonstrating net benefit across clinically relevant threshold probabilities. In practical terms, a threshold probability represents the level of predicted risk at which clinicians may consider whether additional monitoring or multidisciplinary assessment may be appropriate. However, the proposed low-, intermediate-, and high-risk categories were exploratory, have not been externally validated, and should not be used as definitive clinical decision thresholds. For example, patients with predicted risks in the intermediate or high range may prompt clinicians to consider closer maternal laboratory assessment, blood pressure monitoring, fetal surveillance, senior obstetric review, multidisciplinary consultation, neonatal team involvement, and individualized counseling. Nevertheless, these categories should not be used as stand-alone criteria for determining monitoring intensity, intervention, expectant management, or timing of delivery. Decisions regarding expectant management, antenatal corticosteroids, magnesium sulfate administration, transfer to a higher-level care facility, or timing of delivery should continue to be based on comprehensive maternal-fetal assessment, current obstetric guidelines, institutional resources, and individualized clinical judgment.

Despite these strengths, several limitations should be acknowledged. First, this was a retrospective study conducted at a single tertiary teaching hospital in Southwest China, which may limit the external applicability of the model. As a regional referral center for high-risk pregnancies, our hospital may receive a higher proportion of patients with severe EOPE, fetal growth restriction, HELLP syndrome, or other complex maternal-fetal conditions than primary or secondary obstetric institutions. Therefore, the baseline risk, disease spectrum, referral pattern, and management pathway in our cohort may differ from those in community hospitals, non-referral centers, or healthcare systems with different obstetric resources. In addition, differences in population characteristics, ethnicity, socioeconomic status, access to antenatal care, laboratory testing availability, fetal surveillance protocols, maternal-fetal medicine resources, neonatal intensive care capacity, and thresholds for expectant management or delivery may affect the calibration and clinical usefulness of the nomogram in other settings. For this reason, the model should be externally validated in multicenter cohorts and, if necessary, recalibrated before being applied to different populations or healthcare systems. In addition, the exploratory risk thresholds proposed in this study require external validation and potential recalibration, and they should not be interpreted as definitive clinical decision cutoffs.

Second, although internal validation was performed using a split-sample validation cohort and bootstrap resampling was used to generate the bias-corrected calibration curve in the training cohort, full bootstrap-based optimism correction for all model performance measures was not performed. Therefore, the possibility of residual optimism cannot be completely excluded. In addition, although most baseline characteristics were balanced between the training and validation cohorts, the urine protein category remained imbalanced after reclassification. We retained the original prespecified random split to avoid data-driven reassignment, and the urine protein score was included in the prediction model; nevertheless, this imbalance may have affected validation performance and should be considered when interpreting the results. Third, although we defined all predictors according to their availability at admission or within the first 24 h after admission and performed a sensitivity analysis excluding fetal growth restriction and HELLP syndrome, residual temporal misclassification cannot be completely excluded because of the retrospective design. Future prospective studies should define the prediction time point and outcome ascertainment window *a priori* to further minimize this concern. Fourth, some potentially relevant predictors, such as first-trimester aspirin prophylaxis, detailed medication history for autoimmune diseases before or during early pregnancy, blood lipid parameters, angiogenic biomarkers, emerging molecular markers, or advanced imaging parameters, were not included because they were not routinely or consistently available in the retrospective clinical records. In particular, although low-dose aspirin prophylaxis and autoimmune disease treatments may influence the risk and clinical course of preeclampsia, these data were incompletely documented in the inpatient electronic medical records and could not be reliably incorporated into the model without introducing substantial missingness or information bias. Future prospective studies should systematically collect preconception and first-trimester medication histories and evaluate their incremental value for predicting adverse pregnancy outcomes in EOPE. In particular, lipid profiles were not consistently measured at admission in all patients with EOPE during the study period; therefore, including these variables would have increased missingness and may have introduced selection bias. Future studies incorporating such variables may further improve predictive accuracy.

Future research should focus on external validation of the proposed model in multicenter cohorts involving different levels of obstetric care, geographic regions, and healthcare systems. Prospective studies are also warranted to evaluate the clinical impact of implementing the model in routine obstetric practice after adequate external validation and recalibration. In addition, future studies should systematically collect additional laboratory indicators, including blood lipid parameters, angiogenic biomarkers, and emerging molecular markers, to determine whether these variables can further improve the predictive performance and clinical applicability of EOPE risk prediction models. Integrating such biomarkers with routinely available clinical variables, machine learning techniques, and longitudinal clinical data may provide more comprehensive individualized risk assessment in the future.

In conclusion, this study developed and internally validated a nomogram-based prediction model for adverse pregnancy outcomes in women already diagnosed with EOPE. The model is intended for admission-based risk stratification after EOPE diagnosis and should not be interpreted as a tool for early prenatal prediction, population screening, or prediction of the initial occurrence of EOPE. The model demonstrated good discrimination, calibration, and potential clinical utility using routinely available clinical variables. Pending external validation, this prediction tool may assist clinicians in risk stratification and individualized assessment of patients with EOPE at admission.

## Data Availability

The original contributions presented in the study are included in the article/Supplementary material, further inquiries can be directed to the corresponding author/s.

## References

[1] Ali M Ahmed M Memon M Chandio F Shaikh Q Parveen A . Preeclampsia: a comprehensive review. Clin Chim Acta. (2024) 563:119922. doi: 10.1016/j.cca.2024.119922, 39142550

[2] Ngene NC Moodley J. Preventing maternal morbidity and mortality from preeclampsia and eclampsia, particularly in low- and middle-income countries. Best Pract Res Clin Obstet Gynaecol. (2024) 94:102473. doi: 10.1016/j.bpobgyn.2024.102473, 38513504

[3] Kariori M Katsi V Tsioufis C. Late vs. early preeclampsia. Int J Mol Sci. (2025) 26:11091. doi: 10.3390/ijms262211091, 41303573 PMC12652060

[4] Torres-Torres J Espino-Y-Sosa S Martinez-Portilla R Borboa-Olivares H Estrada-Gutierrez G Acevedo-Gallegos S . A narrative review on the pathophysiology of preeclampsia. Int J Mol Sci. (2024) 25:7569. doi: 10.3390/ijms25147569, 39062815 PMC11277207

[5] Rahman L Anwar R Mose JC. Maternal and neonatal outcome among women with early-onset preeclampsia and late-onset preeclampsia. Hypertens Pregnancy. (2024) 43:2405991. doi: 10.1080/10641955.2024.2405991, 39305196

[6] Wang Y Guo X Obore N Ding H Wu C Yu H. Aspirin for the prevention of preeclampsia: a systematic review and meta-analysis of randomized controlled studies. Front Cardiovasc Med. (2022) 9:936560. doi: 10.3389/fcvm.2022.936560, 36440041 PMC9682183

[7] Suksai M Geater A Amornchat P Suntharasaj T Suwanrath C Pruksanusak N. Preeclampsia and timing of delivery: disease severity, maternal and perinatal outcomes. Pregnancy Hypertens. (2024) 37:101151. doi: 10.1016/j.preghy.2024.101151, 39208590

[8] Li X Kang F Li X Du X Yang Y. Comparison of characteristics between early-onset and late-onset severe preeclampsia: a retrospective cohort study from a tertiary Hospital in China. Reprod Sci. (2025) 32:139–49. doi: 10.1007/s43032-024-01674-w, 39134923

[9] Yan M Li F Jun S Li L You W Hu L. Predictive factors for Fetal growth restriction in patients with preeclampsia: a clinical prediction study. Int J Gen Med. (2025) 18:2289–301. doi: 10.2147/IJGM.S510654, 40321938 PMC12047229

[10] Tiruneh SA Vu TTT Moran LJ Callander EJ Allotey J Thangaratinam S . Externally validated prediction models for pre-eclampsia: systematic review and meta-analysis. Ultrasound Obstet Gynecol. (2024) 63:592–604. doi: 10.1002/uog.27490, 37724649

[11] van Eekhout JCA Becking EC Scheffer PG Koutsoliakos I Bax CJ Henneman L . First-trimester prediction models based on maternal characteristics for adverse pregnancy outcomes: a systematic review and meta-analysis. BJOG. (2025) 132:243–65. doi: 10.1111/1471-0528.17983, 39449094 PMC11704081

[12] Wang H Ding Y Zhuang M Li K Zhao S Li D. Application and progress of nomograms in gastric cancer. Front Med. (2025) 12:1510742. doi: 10.3389/fmed.2025.1510742, 39944483 PMC11813919

[13] Domínguez Del Olmo P Herraiz I Villalaín C Galindo A Ayala JL. Predictive modeling of complications arising from early-onset preeclampsia in pregnant women. Womens Health. (2025) 21:17455057251348978. doi: 10.1177/17455057251348978, 40686311 PMC12280542

[14] Croke L. Gestational hypertension and preeclampsia: a practice Bulletin from ACOG. Am Fam Physician. (2019) 100:649–50. 31730305

[15] Magee LA Brown MA Hall DR Gupte S Hennessy A Karumanchi SA . The 2021 International Society for the Study of hypertension in pregnancy classification, diagnosis & management recommendations for international practice. Pregnancy Hypertension. (2022) 27:148–69. doi: 10.1016/j.preghy.2021.09.008, 35066406

[16] Committee Opinion No. 644: The Apgar Score. Obstet Gynecol. (2015) 126:e52–5. doi: 10.1097/AOG.0000000000001108 26393460

[17] Chiang YT Seow KM Chen KH. The pathophysiological, genetic, and hormonal changes in preeclampsia: a systematic review of the molecular mechanisms. Int J Mol Sci. (2024) 25:4532. doi: 10.3390/ijms25084532, 38674114 PMC11050545

[18] Yılmaz M Erdemci F Aşır F Taş F Korak T Aşır A . Role of HMGB1 on the onset of preeclampsia. BMC Pregnancy Childbirth. (2025) 25:626. doi: 10.1186/s12884-025-07701-1, 40442700 PMC12121234

[19] Tadese M Damesa WA Solomon GS Wakie GE Tessema SD Endale A. Maternal outcomes of pre-eclampsia with severe features and its determinants at Abebech Gobena mothers and Childrens health and Saint Peter's specialized hospital, Addis Ababa, Ethiopia: a cross-sectional study. BMJ Open. (2024) 14:e081901. doi: 10.1136/bmjopen-2023-081901, 38553084 PMC10982730

[20] Melesse MF Aynalem GL Badi MB Aynalem BY. Maternal outcomes of severe preeclampsia and eclampsia and associated factors among women admitted at referral hospitals of Amhara regional state, institutional-based cross-sectional study. North-West Ethiopia Front Glob Womens Health. (2025) 6:1555778. doi: 10.3389/fgwh.2025.1555778, 40213383 PMC11983513

[21] Sharma DD Chandresh NR Javed A Girgis P Zeeshan M Fatima SS . The Management of Preeclampsia: a comprehensive review of current practices and future directions. Cureus. (2024) 16:e51512. doi: 10.7759/cureus.51512, 38304688 PMC10832549

[22] Martini C Saeed Z Simeone P Palma S Ricci M Arata A . Preeclampsia: insights into pathophysiological mechanisms and preventive strategies. Am J Prev Cardiol. (2025) 23:101054. doi: 10.1016/j.ajpc.2025.101054, 40703703 PMC12284657

[23] Gilboa I Gabbai D Yogev Y Dominsky O Berger Y Kupferminc M . A prediction model for hemolysis, elevated liver enzymes and low platelets syndrome in pre-eclampsia with severe features. Int J Gynaecol Obstet. (2025) 168:230–6. doi: 10.1002/ijgo.15848, 39118476 PMC11649887

[24] Chahine KM Shepherd MC Sibai BM. Association of Subcapsular Liver Hematoma with Preeclampsia, eclampsia, or Hemolysis, elevated liver enzymes, and low platelet count syndrome. Obstet Gynecol. (2025) 145:335–42. doi: 10.1097/AOG.0000000000005819 39787605

[25] de Ganzo ST de Paco MC Plasencia W. Spiral, uterine artery doppler and placental ultrasound in relation to preeclampsia. Best Pract Res Clin Obstet Gynaecol. (2024) 92:102426. doi: 10.1016/j.bpobgyn.2023.102426, 38039843

[26] Zhang J Wang P Liu J. Clinical value of renal function markers combined with blood lipid levels during late pregnancy to predict preeclampsia: a retrospective case-control study. Clin Exp Obstet Gynecol. (2025) 52:26863. doi: 10.31083/CEOG26863

[27] Mecacci F Romani E Clemenza S Zullino S Avagliano L Petraglia F. Early Fetal growth restriction with or without hypertensive disorders: a clinical overview. Reprod Sci. (2024) 31:591–602. doi: 10.1007/s43032-023-01330-9, 37684516

[28] Wiegel RE Baker K Calderon-Toledo C Gomez R Gutiérrez-Cortez S Houck JA . Impaired maternal central hemodynamics precede the onset of vascular disorders of pregnancy at high altitude. Am J Physiol Heart Circ Physiol. (2025) 328:H174–85. doi: 10.1152/ajpheart.00520.2024, 39657993 PMC11901344

[29] Tedyanto CP Prasetyadi FOH Dewi S Noorlaksmiatmo H. Maternal factors and perinatal outcomes associated with early-onset versus late-onset fetal growth restriction: a meta-analysis. J Matern Fetal Neonatal Med. (2025) 38:2505774. doi: 10.1080/14767058.2025.2505774, 40374573

[30] Arnouts L Terwingen J Van Damme K Mannaerts D. The added value of the sFlt-1/PlGF ratio in pregnant women with intrauterine growth restriction (IUGR) with or without preeclampsia on adverse pregnancy outcomes and neonatal morbidities: a retrospective study. BMC Pregnancy Childbirth. (2025) 25:959. doi: 10.1186/s12884-025-08057-2, 41029220 PMC12486873

[31] Liauw J Gordijn SJ Ganzevoort W Mayer C Hutcheon JA. Antenatal diagnosis of early-onset small for gestational age: absolute and relative risks of adverse outcomes. Am J Obstet Gynecol. (2025) 233:329.e1–329.e14. doi: 10.1016/j.ajog.2025.04.041, 40262728

[32] von Dadelszen P Payne B Li J Ansermino JM Broughton Pipkin F Côté AM . Prediction of adverse maternal outcomes in pre-eclampsia: development and validation of the fullPIERS model. Lancet. (2011) 377:219–27. doi: 10.1016/S0140-6736(10)61351-7, 21185591

[33] Payne BA Hutcheon JA Ansermino JM Hall DR Bhutta ZA Bhutta SZ . A risk prediction model for the assessment and triage of women with hypertensive disorders of pregnancy in low-resourced settings: the miniPIERS (pre-eclampsia integrated estimate of RiSk) multi-country prospective cohort study. PLoS Med. (2014) 11:e1001589. doi: 10.1371/journal.pmed.1001589, 24465185 PMC3897359

[34] Thangaratinam S Allotey J Marlin N Dodds J Cheong-See F von Dadelszen P . Prediction of complications in early-onset pre-eclampsia (PREP): development and external multinational validation of prognostic models. BMC Med. (2017) 15:68. doi: 10.1186/s12916-017-0827-3, 28356148 PMC5372261

