# Supplementary material for: Development and internal validation of a nomogram for predicting adverse pregnancy outcomes in women with early-onset preeclampsia
Source: Front Med (Lausanne). 2026 Jun 17;13:1828294. doi: 10.3389/fmed.2026.1828294 (PMC13318994; doi:10.3389/fmed.2026.1828294)
Supplement: Supplementary file 1 [file Table_1.DOCX]

**Supplementary Table S1.** Definition and classification of composite adverse pregnancy outcome components

| **Component** | **Classification** | **Diagnostic definition** | **Reference/criteria** |
| --- | --- | --- | --- |
| Eclampsia | Maternal | New-onset generalized tonic–clonic seizures in a woman with preeclampsia, not attributable to other neurological causes. | ACOG/ISSHP criteria |
| Placental abruption | Maternal/fetal | Premature separation of the placenta diagnosed based on clinical symptoms, ultrasonographic findings, intraoperative findings, or placental examination. | Standard obstetric clinical criteria |
| Pulmonary edema | Maternal | Acute pulmonary edema diagnosed based on respiratory symptoms, oxygen desaturation, chest imaging, and treatment records. | ACOG/ISSHP criteria |
| Acute kidney injury | Maternal | Newly developed renal dysfunction during hospitalization, based on increased serum creatinine levels and clinical diagnosis by the treating obstetric team. | ACOG/ISSHP criteria |
| Disseminated intravascular coagulation | Maternal | Coagulation dysfunction diagnosed according to clinical bleeding manifestations and abnormal coagulation test results documented in the medical record. | Standard obstetric clinical criteria |
| Intensive care unit admission | Maternal | Transfer to the intensive care unit due to severe maternal complications or requirement for intensive monitoring or organ support. | Medical record documentation |
| Fetal distress requiring urgent delivery | Fetal/neonatal | Non-reassuring fetal status documented by fetal monitoring, Doppler assessment, or obstetric evaluation leading to urgent delivery. | Standard obstetric clinical criteria |
| Intrauterine fetal death or stillbirth | Fetal/neonatal | Fetal death occurring before or during delivery, documented in obstetric or delivery records. | Standard obstetric clinical criteria |
| Neonatal compromised condition requiring immediate resuscitation | Fetal/neonatal | A 5-minute Apgar score <7 and/or requirement for immediate neonatal resuscitation documented by the attending neonatologist. | ACOG/AAP neonatal assessment guidance |
| Neonatal death | Fetal/neonatal | Death of a live-born neonate during the early neonatal period as documented in neonatal records. | Neonatal record documentation |

**Note:** The composite adverse pregnancy outcome was defined as the occurrence of at least one predefined maternal or fetal/neonatal adverse event after admission and during hospitalization, delivery, or the early neonatal period. The selection and definition of outcome components were informed by authoritative guidance on hypertensive disorders of pregnancy and neonatal assessment, including ACOG guidance, ISSHP 2021 recommendations, and ACOG/AAP guidance on Apgar score interpretation. Fetal growth restriction, oligohydramnios, and HELLP syndrome identified at admission or during the initial clinical evaluation were considered baseline pregnancy-related complications or candidate predictors and were not included as components of the composite adverse pregnancy outcome, to avoid overlap between predictors and outcomes. ACOG = American College of Obstetricians and Gynecologists; AAP = American Academy of Pediatrics; ISSHP = International Society for the Study of Hypertension in Pregnancy; HELLP = hemolysis, elevated liver enzymes, and low platelet syndrome.

**Supplementary Table S2.** Performance of the sensitivity model excluding fetal growth restriction and HELLP syndrome

| **Cohort** | **Predictors included** | **AUC** | **95% CI** | **Hosmer–Lemeshow χ²** | **Hosmer–Lemeshow P value** | **Calibration intercept** | **Calibration slope** |
| --- | --- | --- | --- | --- | --- | --- | --- |
| Training cohort | Gestational age at admission, systolic blood pressure, urine protein score, platelet count, and AST level | 0.778 | 0.723–0.832 | 7.23 | 0.513 | 0.000 | 1.000 |
| Validation cohort | Gestational age at admission, systolic blood pressure, urine protein score, platelet count, and AST level | 0.722 | 0.630–0.809 | 17.98 | 0.021 | 0.256 | 0.741 |

**Note:** The sensitivity model excluded fetal growth restriction and HELLP syndrome to assess the robustness of the primary prediction model and to address the potential concern regarding circularity between baseline pregnancy-related complications and the composite adverse pregnancy outcome. AUC = area under the receiver operating characteristic curve; AST = aspartate aminotransferase; HELLP = hemolysis, elevated liver enzymes, and low platelet syndrome.

**Supplementary Table S3.** Missing-data proportions for candidate predictors and the composite adverse pregnancy outcome

| **Variable** | **Missing values, n** | **Missing proportion, %** |
| --- | --- | --- |
| Maternal age | 0 | 0.0 |
| Pre-pregnancy BMI | 0 | 0.0 |
| Primipara | 0 | 0.0 |
| Chronic hypertension | 0 | 0.0 |
| Pregestational diabetes | 0 | 0.0 |
| Gestational age at admission | 0 | 0.0 |
| Systolic blood pressure | 0 | 0.0 |
| Diastolic blood pressure | 0 | 0.0 |
| Urine protein score | 0 | 0.0 |
| Platelet count | 0 | 0.0 |
| AST | 0 | 0.0 |
| ALT | 0 | 0.0 |
| Serum creatinine | 0 | 0.0 |
| Uric acid | 0 | 0.0 |
| LDH | 0 | 0.0 |
| Hemoglobin | 0 | 0.0 |
| Fetal growth restriction | 0 | 0.0 |
| Oligohydramnios | 0 | 0.0 |
| HELLP syndrome | 0 | 0.0 |
| Magnesium sulfate administration | 0 | 0.0 |
| Composite adverse pregnancy outcome | 0 | 0.0 |

**Note:** Missing data were assessed in the final analytic dataset of 425 eligible patients. No missing values were observed for any candidate predictor or outcome variable. Therefore, complete-case analysis was performed, and multiple imputation was not required. AST = aspartate aminotransferase; ALT = alanine aminotransferase; BMI = body mass index; HELLP = hemolysis, elevated liver enzymes, and low platelet syndrome; LDH = lactate dehydrogenase.

**Supplementary Table S4.** Candidate variables considered for model development and rationale for inclusion or exclusion

| **Variable** | **Entered into LASSO model** | **Rationale** |
| --- | --- | --- |
| Maternal age | Yes | Routinely available demographic variable at admission; clinically relevant to pregnancy outcomes. |
| Pre-pregnancy BMI | Yes | Routinely recorded maternal characteristic; associated with hypertensive disorders and pregnancy outcomes. |
| Parity | Yes | Routinely available obstetric history variable. |
| Chronic hypertension | Yes | Preexisting medical condition associated with preeclampsia severity and adverse outcomes. |
| Pregestational diabetes | Yes | Preexisting medical condition potentially associated with maternal and fetal complications. |
| Gestational age at admission | Yes | Admission-based obstetric variable reflecting disease timing and fetal maturity. |
| Systolic blood pressure | Yes | Admission clinical parameter reflecting disease severity. |
| Diastolic blood pressure | Yes | Admission clinical parameter reflecting disease severity. |
| Urine protein score | Yes | Admission urinalysis variable reflecting renal involvement. |
| Platelet count | Yes | First available laboratory indicator after admission; reflects hematologic involvement. |
| AST | Yes | First available laboratory indicator after admission; reflects hepatic involvement. |
| ALT | Yes | First available laboratory indicator after admission; reflects hepatic involvement. |
| Serum creatinine | Yes | First available laboratory indicator after admission; reflects renal involvement. |
| Uric acid | Yes | First available laboratory indicator after admission; associated with placental dysfunction and disease severity. |
| LDH | Yes | First available laboratory indicator after admission; reflects hemolysis or tissue injury. |
| Hemoglobin | Yes | First available laboratory indicator after admission; routinely available hematologic variable. |
| Fetal growth restriction | Yes | Baseline pregnancy-related complication diagnosed at admission or during initial evaluation; reflects placental dysfunction and fetal compromise; not counted as an outcome component. |
| Oligohydramnios | Yes | Baseline pregnancy-related complication diagnosed at admission or during initial evaluation; reflects possible placental insufficiency and fetal risk; not counted as an outcome component. |
| HELLP syndrome | Yes | Baseline pregnancy-related complication diagnosed at admission or during initial evaluation; reflects maternal hematologic and hepatic involvement; not counted as an outcome component. |
| First-trimester low-dose aspirin prophylaxis | No | Excluded a priori because first-trimester outpatient medication history was not consistently documented in the retrospective inpatient records for all patients; inclusion would have introduced substantial missingness and information bias. |
| Medication history for autoimmune diseases before or during early pregnancy | No | Excluded a priori because detailed medication exposure before and during early pregnancy was not consistently available in the retrospective records; patients with documented autoimmune diseases requiring long-term systemic treatment that could substantially affect pregnancy outcomes were excluded according to the eligibility criteria. |
| Blood lipid parameters, including triglycerides, total cholesterol, HDL-C, and LDL-C | No | Excluded a priori because lipid profiles were not routinely measured at admission in all patients with EOPE during the study period; inclusion would have introduced substantial missingness and potential selection bias. |
| Magnesium sulfate administration | No | Excluded a priori because it represents treatment after clinical assessment and may reflect disease management rather than baseline risk. |
| Delivery mode | No | Excluded a priori because it occurs after prediction and may be influenced by disease progression or clinical decision-making. |
| Gestational age at delivery | No | Excluded a priori because it occurs after prediction and may be part of the clinical pathway following risk assessment. |
| Neonatal birth weight | No | Excluded a priori because it is unavailable at admission and occurs after the prediction time point. |
| Neonatal intensive care unit admission | No | Excluded a priori because it is an outcome-related variable occurring after delivery. |
| Components of the composite adverse pregnancy outcome | No | Excluded a priori to avoid circularity and post-outcome information. |

**Note:** Candidate predictors were restricted to variables measured or diagnosed at admission or within the first 24 hours after admission, before the occurrence of the predefined composite adverse pregnancy outcome. First-trimester low-dose aspirin prophylaxis, early-pregnancy medication history for autoimmune diseases, and blood lipid parameters were considered potentially relevant but were not included in the LASSO model because they were not consistently available in the retrospective inpatient records. Patients with documented autoimmune diseases requiring long-term systemic treatment that could substantially affect pregnancy outcomes were excluded according to the eligibility criteria. AST = aspartate aminotransferase; ALT = alanine aminotransferase; BMI = body mass index; EOPE = early-onset preeclampsia; HDL-C = high-density lipoprotein cholesterol; HELLP = hemolysis, elevated liver enzymes, and low platelet syndrome.

**Supplementary Table S5.** Structured comparison between the present nomogram and existing prediction tools for preeclampsia-related adverse outcomes

| **Model/tool** | **Target population** | **Main outcome** | **Key predictors** | **Validation status** | **Main applicability** |
| --- | --- | --- | --- | --- | --- |
| fullPIERS | Women with preeclampsia | Adverse maternal outcomes within a short-term risk window | Gestational age, chest pain or dyspnea, oxygen saturation, platelet count, serum creatinine, AST | Developed and validated in multicenter cohorts | Maternal risk assessment and short-term triage in preeclampsia |
| miniPIERS | Women with hypertensive disorders of pregnancy, particularly in low-resource settings | Maternal death or major hypertensive-related complications | Simple clinical and symptom-based variables, with or without pulse oximetry | Developed in a multicountry prospective cohort | Triage and risk assessment where laboratory resources may be limited |
| PREP-L/PREP-S | Women with early-onset preeclampsia | Adverse maternal outcomes by discharge or over time after diagnosis | Maternal characteristics, clinical signs, blood pressure, urine protein, laboratory variables, and disease-severity indicators | Developed and externally validated in early-onset preeclampsia cohorts | Individualized maternal risk estimation and triage in EOPE |
| Recent EOPE-specific prediction models | Women with EOPE | Maternal complications or EOPE-related adverse outcomes | Clinical, laboratory, and sometimes advanced computational features | Variable; often limited external validation | Exploratory individualized prediction and model comparison |
| Present nomogram | Women with EOPE admitted to a tertiary hospital in Southwest China | Composite adverse pregnancy outcome including maternal and fetal/neonatal events | Gestational age at admission, systolic blood pressure, urine protein score, platelet count, AST, fetal growth restriction, HELLP syndrome | Internally validated using split-sample validation; external validation not yet performed | Admission-based risk stratification for maternal-fetal monitoring and individualized management |

**Note:** The present nomogram differs from established models mainly in its outcome definition, which includes both maternal and fetal/neonatal adverse events, and in its development population. It should be considered an internally validated risk-stratification tool that requires external validation before routine clinical implementation. AST = aspartate aminotransferase; EOPE = early-onset preeclampsia; HELLP = hemolysis, elevated liver enzymes, and low platelet syndrome; PREP = Prediction of Risks in Early-onse
